# Risk Factors Associated with a Dengue Fever Outbreak in Islamabad, Pakistan: Case-Control Study

**DOI:** 10.2196/27266

**Published:** 2021-12-30

**Authors:** Amjad Mehmood, Fawad Khalid Khan, Ambreen Chaudhry, Zakir Hussain, Mumtaz Ali Laghari, Ijaz Shah, Zeeshan Iqbal Baig, Mirza Amir Baig, Yousef Khader, Aamer Ikram

**Affiliations:** 1 Field Epidemiology and Laboratory Training Program Islamabad Pakistan; 2 Department of Public Health Jordan University of Science and Technology Irbid Jordan; 3 National Institute of Health Pakistan Islamabad Pakistan

**Keywords:** dengue fever, DENV-2, outbreak investigation, Islamabad, Pakistan, outbreak, epidemiology, disease surveillance, surveillance, vector

## Abstract

**Background:**

On October 23, 2016, 79 dengue fever cases were reported from the Union Council Tarlai to Federal Disease Surveillance and Response Unit Islamabad. A team was established to investigate the suspected dengue outbreak.

**Objective:**

The aim of this study was to determine the extent of the outbreak and identify the possible risk factors.

**Methods:**

Active case finding was performed through a house-to-house survey. A case was defined as an acute onset of fever ≥38℃ in a resident of Tarlai from October 2 to November 11, 2016, with a positive dengue virus (nonstructural protein, NS-1) test and any of the two of following signs and symptoms: retroorbital/ocular pain, headache, rash, myalgia, arthralgia, and hemorrhagic manifestations. A structured questionnaire was used to collect data. Age- and sex-matched controls (1:1) were identified from residents in the same area as cases. Blood samples were taken and sent to the National Institute of Health for genotype identification.

**Results:**

During the active case search, 145 cases of dengue fever were identified by surveying 928 houses from October 23 to November 11, 2016. The attack rate (AR) was 17.0/10,000. The mean age was 34.4 (SD 14.4) years. More than half of the cases were male (80/145, 55.2%). Among all cases, 29% belonged to the 25-34 years age group and the highest AR was found in the 35-44 years age group (35.6/10,000), followed by the 55-64 years age group (35.5/10,000). All five blood samples tested positive for NS-1 (genotype DENV-2). The most frequent presenting signs/symptoms were fever and headache (both 100%). Stagnant water around houses (odds ratio [OR] 4.86, 95% CI 2.94-8.01; *P*<.001), presence of flower pots in the home (OR 2.73, 95% CI 1.67-4.45; *P*<.001), and open water containers (OR 2.24, 95% CI 1.36-3.60; *P*<.001) showed higher odds among cases. Conversely, use of bed nets (OR 0.44, 95% CI 0.25-0.77; *P*=.003), insecticidal spray (OR 0.33, 95% CI 0.22-0.55; *P*<.001), door screens (OR 0.27, 95% CI 0.15-0.46; *P*<.001), mosquito coil/mat (OR 0.26, 95% CI 0.16-0.44; *P*<.001), and cleanliness of the house (OR 0.12, 95% CI 0.05-0.26; *P*<.001) showed significant protective effects.

**Conclusions:**

Stagnant water acting as breeding grounds for vectors was identified as the probable cause of spread of the dengue outbreak. Establishment of surveillance and an early reporting system along with use of protective measures against the vector are strongly recommended.

## Introduction

Dengue fever is a vector-borne viral disease transmitted by *Aedes aegypti* and *Aedes albopictus* [1] carrying dengue virus, which is a single-stranded RNA virus from the *Flaviviridae* family [2]. There are four distinct serotypes of dengue virus: DEN-1, DEN-2, DEN-3, and DEN-4 [3]. Recovery from infection by dengue virus provides lifelong immunity against that particular virus serotype. However, this immunity confers only partial and transient protection against subsequent infections by the other three serotypes of the virus. If the same individual is infected with another serotype, they can become seriously ill and develop complications of dengue hemorrhagic fever (DHF) or dengue shock syndrome (DSS) [4]. Clinical features include flu-like symptoms with high-grade fever, headaches, nausea, vomiting, body aches, retroorbital pain, swollen glands/joints, bone or muscle pain, and rash [5].

Since 1970, the upsurge in all dengue virus serotypes has increased the danger of severe disease pertaining to secondary infections, resulting in steady growth in the frequency of epidemics [6]. During the last two decades of the 19th century and in the first two decades of the 20th century, dengue became sporadic in tropical and subtropical countries of the world [7]. Worldwide, the incidence is 50-100 million dengue cases and 250,000-500,000 cases of DHF per year. DHF/DSS is associated with a mortality rate up to 5%-10%. Dengue fever affects both genders; however, males are predominantly more affected [8,9].

In Pakistan, the first outbreak of dengue fever was reported from Karachi in 1994. Subsequently, huge outbreaks were reported from Karachi in 2005, Lahore in 2011, and Swat in 2013 with 6376 cases and 23 deaths. DEN-2 and DEN-3 were identified as the most prevalent serotypes in Pakistan [9]. Research has shown that a humid/warm environment favors the breeding of the mosquito vectors [10]. Various studies have also reported certain risk factors for dengue fever. Small puddles of stagnant water in plants, tires, and ditches have been identified as the preferred breeding sites of vectors. Clothing with long sleeves and legs, use of mosquito repellent, and vector control (insecticide residual spray and thermal fogging) have been determined to be the most effective preventive measures against dengue fever.

On October 23, 2016, the medical officer of a first-level health care facility (Basic Health Unit) in Union Council (UC) Tarlai telephonically communicated with the Federal Disease Surveillance and Response Unit at the National Institute of Health, Islamabad, that he had witnessed 79 dengue cases in only 3 days. On the same day, a team comprising members of the Field Epidemiology and Laboratory Training Program along with the local medical officer was deployed to investigate the dengue outbreak. The aim of this study was to determine the extent of the outbreak and identify the possible risk factors responsible for spread of the disease.

## Methods

The investigation team performed a descriptive study and identified all cases that presented with fever. A case-control study was then performed from October 23 to November 11, 2016. Active cases, along with the cases reported earlier, were enrolled through a house-to-house case search. Diagnostic criteria set by the district government were used to enroll the cases in which a dengue virus antigen detection test targeting nonstructural protein-1 (NS-1) was used as a confirmatory test.

A case was defined as acute onset of fever ≥38℃ in a resident of Tarlai from October 2, 2016, to November 11, 2016, with a positive NS-1 test and any two of the following signs and symptoms: retroorbital pain, headache, rash, myalgia, arthralgia, and hemorrhagic manifestations (petechial spots, purpura, bleeding from the gums or nose).

Age- and sex-matched controls were selected from the neighborhood of the cases. Controls were residents who did not have fever and associated signs and symptoms, and had not tested positive for NS-1 during this time period. Information from inhabitants of the area, fitting the case definition, was collected using a structured questionnaire. Information was collected on demographics; dates of onset of illness, sign/symptoms, and laboratory confirmation; along with other relevant possible risk factors. A line list was prepared and a total of five blood samples were taken from the patients of UC Tarlai for laboratory confirmation.

Data were analyzed using Epi-info version 7. The mean age was calculated. Age groups were created to compute age group–specific attack rates (ARs). Frequencies of each variable were calculated. Multivariate analysis was performed to determine the risk factors. A *P* value <.05 was considered statistically significant.

## Results

### Patient Characteristics

During the active case search, 145 cases of dengue fever were identified by surveying 928 houses from October 23 to November 11, 2016. The overall AR was 17.0/10,000 (the total population of UC Tarlai was 84,810 during the study period). The mean age of the cases was 34.4 (SD 14.4) years (range 6-80 years). More than half of the cases (80/145, 55.2%) were men. Approximately 30% of the cases belonged to the age group 25-34 years, followed by 35-44 years, and 15-24 years. The age-specific AR per 10,000 individuals in the population showed the highest incidence among the 35-44 years age group, followed by the 55-64 years age group (Table 1).

**Table 1:** Age distribution of dengue fever cases among residents of Union Council Tarlai, Islamabad, from October 23 to November 11, 2016 (N=145).

Age group (years)	Cases, n (%)	Population, n	Attack rate (per 10,000)
<5	0 (0)	11,024	0.00
5-14	10 (6.9)	22,975	4.3
15-24	26 (17.9)	17,836	14.5
25-34	42 (29.0)	12,602	33.3
35-44	31 (21.4)	8700	35.6
45-54	20 (13.8)	5808	34.4
55-64	12 (8.3)	3374	35.5
65-74	2 (1.4)	1703	17.7
≥75	2 (1.4)	788	25.3

### Signs and Symptoms

As shown in Table 2, the most frequent signs/symptoms were fever and headache, followed by myalgia and joint/bone pain, whereas only few patients presented with mucosal bleeding. Five blood samples were taken from the patients; all were positive for dengue fever (NS-1 antigen) and one was identified as genotype DEN-2.

**Table 2:** Clinical presentation of dengue fever cases in Union Council Tarlai, Islamabad, from October 23 to November 11, 2016 (N=145).

Signs and symptoms	Cases, n (%)
Fever	145 (100.0)
Headache	145 (100.0)
Myalgia	122 (84.1)
Joint/bone pain	121 (83.4)
Retroorbital pian	113 (77.9)
Nausea/vomiting	113 (77.9)
Abdominal pain	90 (62.1)
Petechia	64 (44.1)
Impaired consciousness	32 (22.1)
Rash	24 (16.6)
Mucosal bleeding	8 (5.5)

### Preventive Measures and Distribution of Risk Factors

The use of different preventive measures was determined from the surveys with the 145 dengue fever cases. The majority of patients wore full cloth for protection against mosquito bites, approximately half used insecticide spray or a mosquito coil/mat stand, a third used repellent lotion, and bed net use was the least frequent preventive measure adopted. The possible risk factors were stagnant water around the house, presence of flower pots in the house, and open containers of water in the house (Table 3).

**Table 3:** Distribution of preventive measures adopted and possible risk factors among 145 dengue fever cases in Union Council Tarlai, Islamabad, from October 23 to November 11, 2016.

Variables		Cases, n (%)
**Preventive measures**		
	Use of full clothing	129 (89.9)
	Presence of door screening	82 (56.6)
	Use of insecticide spray	80 (55.2)
	Use of mosquito coil/mat	72 (49.7)
	Use of repellent lotion	48 (33.1)
	Use of bed net	24 (16.6)
**Possible risk factors**		
	Stagnant water around house	106 (73.1)
	Presence of flower pots in house	74 (51.0)
	Open container of water in house	63 (43.4)
	Uncleaned house	47 (32.4)
	Water pools	22 (15.2)
	Old tires in or around house	14 (11.2)
	Travel history	16 (11.3)
	History of blood donation	0 (0)

### Epidemiologic Curve

**Figure 1:**
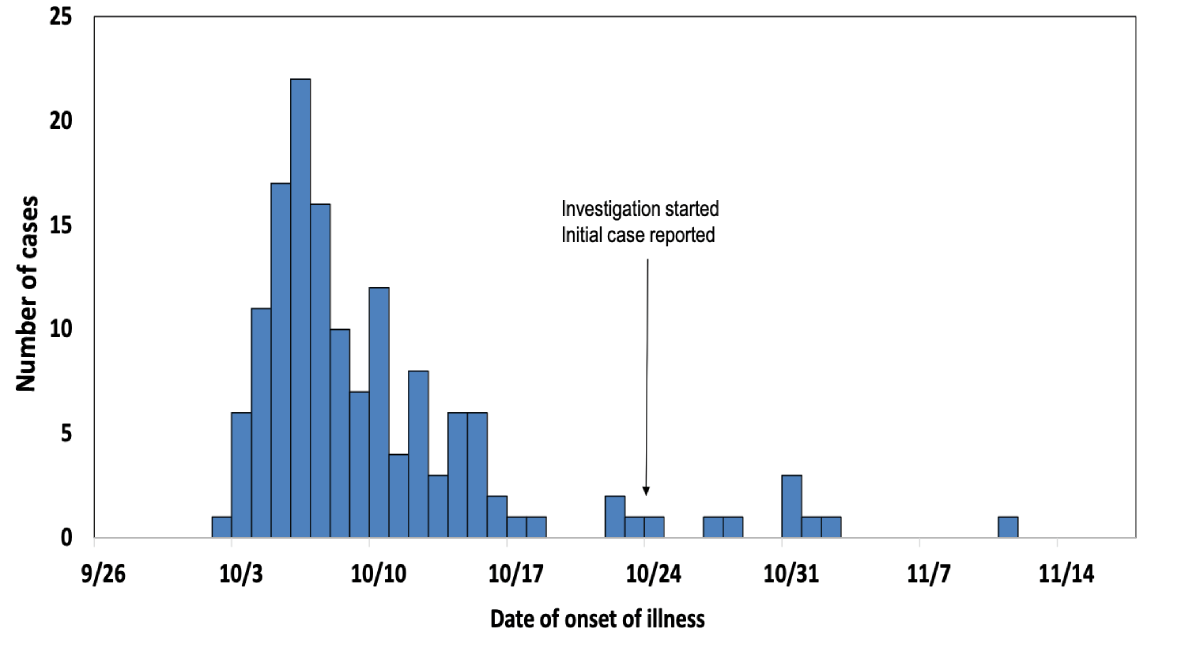
Epidemic curve. Number of cases by the date of onset of illness (month/year) during the dengue fever outbreak in Union Council Tarlai, Islamabad (October 23 to November 11, 2016).

The epidemiologic curve showed that the first case had a date of onset of illness of October 2, 2016, and most of the cases developed signs/symptoms on October 6 (n=22), followed by October 5 and October 7 (Figure 1).

Approximately 99 mm of rain fell on September 1, with two additional rain showers occurring in the middle of September.

### Factors Associated With Dengue Fever

In the bivariate analysis, stagnant water around houses, use of flower pots, and open water containers were significantly associated with increased odds of dengue fever among cases. Use of bed nets, insecticidal spray, a door screen, and a mosquito coil/mat, along with cleanliness in the house showed significant protective effects (Table 4).

On multivariate logistic regression, stagnant water (adjusted odds ratio 4.25, 95% CI 2.42-7.46, *P*<.001) and open containers in the house (adjusted odds ratio 1.94, CI 1.09-3.42, *P*=.02) showed significant associations with dengue fever incidence.

**Table 4:** Bivariate analysis of factors associated with dengue fever in Tarlai, Islamabad (October 23 to November 11, 2016).

Factor	Cases, n	Controls, n	OR^a^	95% CI	*P* value
Stagnant water	106	52	4.86	2.94-8.01	<.001
Flower pots	74	40	2.73	1.67-4.45	<.001
Open water container	63	37	2.24	1.36-3.60	<.001
Use of bed nets	24	45	0.44	0.25-0.77	.003
Insecticide spray	80	114	0.33	0.20-0.55	<.001
Door screen	82	120	0.27	0.15-0.46	<.001
Use of mosquito coil/mat	72	114	0.26	0.16-0.44	<.001
Cleanliness	98	137	0.12	0.05-0.26	<.001
Water pool	22	11	2.17	1.01-4.67	.04
Use of full clothing	129	139	0.34	0.13-0.91	.03

^a^OR: odds ratio.

## Discussion

### Principal Findings

Dengue fever is one of the most rapidly spreading diseases in the world, with a 30-fold increase in incidence in the last 50 years [11]. In this study, a dengue fever outbreak was investigated in a rural area of Islamabad capital territory. This study showed that the number of males with dengue fever exceeded the number of females affected by dengue fever. This finding is consistent with findings of another study [12] and might be explained by the fact that males are more involved in outdoor activities in Pakistani culture and hence are more prone to infection. Moreover, the extreme age groups (under 14 and above 65 years) were less commonly affected as compared to the working age groups, which is attributed to the fact that the former age groups tend to stay at home most of the time. This finding supports the conclusions of a similar study performed in Swat [9].

Meteorological data showed that the local clustering of cases is likely due to the accumulation of stagnant water that facilitates the endemic vector species *Aedes aegypti* to breed. Strong associations of dengue fever with the presence of stagnant water around houses and flower pots in houses were determined. Stagnant water was previously identified as a risk factor in another outbreak in Lahore, Pakistan [13]. However, this study in Lahore in 2012 showed that dengue fever was associated with the piped water supply, which is in contrast to our findings. Open fields and empty plots are the main sites for accumulation of stagnant water that become breeding sites for mosquitoes. Similarly, accumulated rainwater around houses and uncovered receptacles serve as important breeding grounds for these vectors.

**Figure 2:**
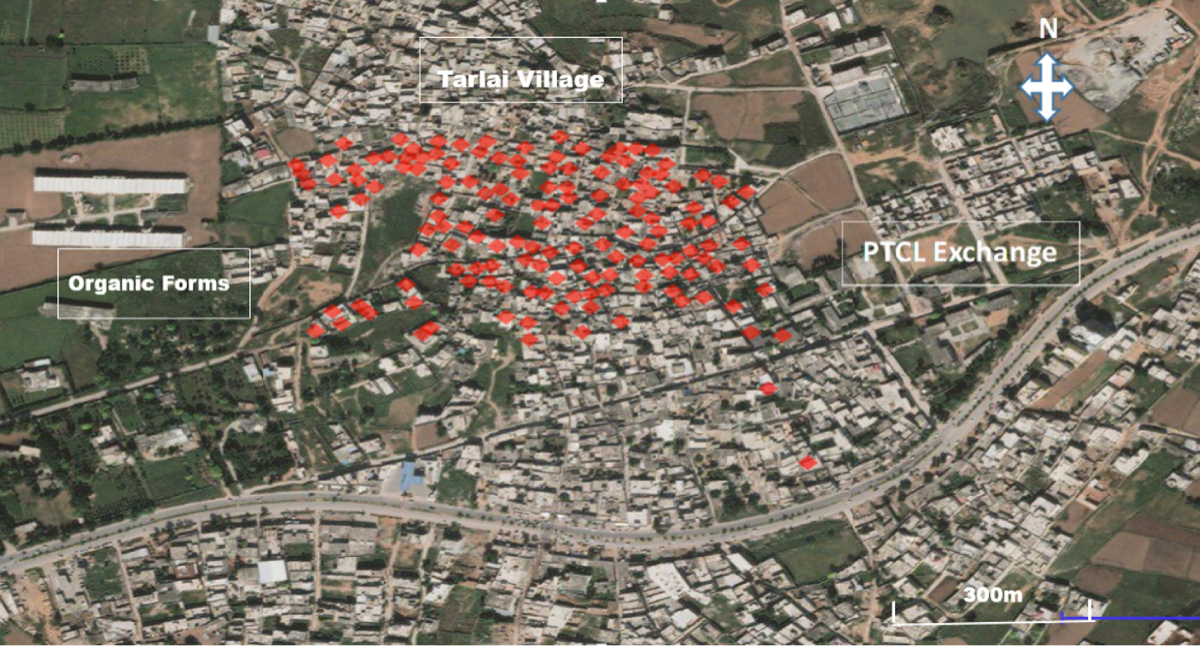
Spot map of Union Council Tarlai, Islamabad, during the dengue fever outbreak from October 23 to November 11, 2016.

A detailed environmental survey is called upon to understand the mechanism of hatching of larvae after the first rain, in which mosquitos reach the adult stage and are therefore capable of disease transmission. Our findings showed that cases clusters in the village (Figure 2), suggesting a link with previous rainfall at the start of September and October. Therefore, routine entomological surveillance for dengue virus is of great importance to enable early detection of spatial and temporal links of the outbreaks.

The NS-1 test is widely used for diagnostic purposes [14]. A positive NS-1 test has equal sensitivity as real-time polymerase chain reaction in the early phase of disease; however, the NS-1 test is a cheaper and more readily available tool for the early diagnosis of dengue [13]. Previously reported outbreaks showed the presence of the DEN-2 and DEN-3 genotypes, which is consistent with our finding that DEN-2 was the only serotype identified in the Tarlai outbreak [15]. Only one sample was sent to the public health laboratory for genotyping due to lack of resources, which confirmed the DEN-2 genotype.

The main limitations of this study are the lack of environmental analysis related to vector prevalence and the possibility of recall bias, especially in the control group.

### Conclusion

Stagnant water was significantly associated with a dengue fever outbreak in UC Tarlai, Islamabad, which is the most probable cause for spread of the disease. Therefore, the community should have been educated about the normal habitat of the dengue virus vector. Use of protective measures against the vector is strongly recommended. Different control measures should be implemented, including a media campaign for awareness and health education of vector control (mosquito), capacity building of health staff for timely disease detection and control, proper disposal of solid waste, indoor residual spray/fumigation for larval control, and a flower pot dryness campaign to reduce the indoor breeding sites of mosquitoes. Moreover, awareness campaigns for the use of repellents and protective measures, and establishment of a surveillance system (sanitization of health staff for reporting) for detection of dengue fever are strongly recommended.

## Abbreviations

AR: attack rate
DHF: dengue hemorrhagic fever
DSS: dengue shock syndrome
NS-1: nonstructural protein-1
UC: Union Council
